# Transcriptome profiling of symptomatic vs. asymptomatic grapevine plants reveals candidate genes for plant improvement against trunk diseases

**DOI:** 10.1186/s12870-025-06763-9

**Published:** 2025-07-02

**Authors:** M. Patanita, M. D. Campos, A. Albuquerque, J. A. Ribeiro, D. Pimentel, R. S. Ramiro, T. Monteiro, M. Basaloco, F. Santos, I. Castro, M. R. Félix

**Affiliations:** 1 MED - Mediterranean Institute for Agriculture, Environment and Development & CHANGE - Global Change and Sustainability Institute, Institute for Advanced Studies and Research, Universidade de Évora, Pólo da Mitra, Ap. 94, Évora, 7006-554 Portugal; 2 MED - Mediterranean Institute for Agriculture, Environment and Development & CHANGE - Global Change and Sustainability Institute, Departamento de Fitotecnia, Escola de Ciências e Tecnologia, Universidade de Évora, Pólo da Mitra, Ap. 94, Évora, 7006-554 Portugal; 3 InnovPlantProtect - Associação, Estrada de Gil Vaz, Ap. 72, Elvas, 7350-999 Portugal; 4 Centre for Research and Technology of Agro-Environmental and Biological Sciences (CITAB), Institute for Innovation, Capacity Building and Sustainability of Agri-Food Production (Inov4Agro), Department of Genetics and Biotechnology, University of Trás-os-Montes e Alto Douro, Vila Real, 5000-801 Portugal

**Keywords:** *Vitis vinifera* L., Fungal diseases, Trunk pathogens, Plant response to biotic stress, RNA-seq

## Abstract

**Background:**

Grapevine trunk diseases (GTDs) are a disease complex that pose a serious challenge to vineyard productivity worldwide. Their increasing occurrence, coupled with the absence of effective treatments, turns their incidence one of the biggest obstacles to viticulture. A deeper understanding of grapevine defence mechanisms is essential to develop new strategies for a sustainable disease management.

**Results:**

This study explored the transcriptome profiling of GTDs symptomatic and asymptomatic plants of ‘Trincadeira’ (a slightly susceptible or tolerant cultivar to GTDs) and ‘Alicante Bouschet’ (a highly susceptible susceptible or susceptible cultivar to GTDs), under natural field conditions. RNA-seq yielded 1 598 differentially expressed genes (DEGs) when comparing cultivars, and 64 DEGs associated with symptomatology, regardless of the cultivar. Transport was revealed as the main biological process involved, predominantly activated in ‘Alicante Bouschet’, indicating a possible link between these genes and disease progression. Conversely, the relative tolerance of ‘Trincadeira’ to this disease complex might be supported by the activation of secondary and hormonal metabolism and the differential expression of a set of defence-related genes, which may act as key factors to limit GTDs infection. An important role of the peroxidase gene *PER42* in the inhibition of GTDs symptoms was highlighted in this study.

**Conclusions:**

Our results provide novel insights into grapevine resistance mechanisms to GTDs and highlight candidate genes for improving disease tolerance. To the best of our knowledge, this is the first transcriptomic study of naturally infected grapevines with multiple trunk pathogens under natural field conditions. By modulating the activation or inhibition of key plant response regulators, it may be possible to enhance resistance, offering sustainable and effective strategies for successful management of GTDs.

**Clinical trial number:**

Not applicable.

**Supplementary Information:**

The online version contains supplementary material available at 10.1186/s12870-025-06763-9.

## Background

Crop production losses due to the presence of phytopathogens can go up to 40% every year [1]. This is aggravated by other factors such as the continuously growing world population [2, 3], leading to increased demand for food, and the commitment to reduce by 50% the use of phytopharmaceuticals by 2030, promoted by the European Green Deal’s “Farm to Fork” strategy. To fulfil these global requirements, FAO promotes sustainable and ecological approaches, prompt response, innovative and environmentally friendly preventive control strategies for sustainable disease management [1].

Grapevine (*Vitis vinifera* L.) is one of the world’s most commercially important perennial crops, with a total area of 7.2 million hectares and a world wine production estimated at 237 million hectolitres in 2023, the smallest in the last 60 years [4]. Portugal is currently among the ten major wine producers in the world and is a prominent wine exporter, with 7.5 million hectolitres produced, in a total vineyard area of 182 kha [4]. Grapevine plants are threatened by a wide range of diseases, in which grapevine trunk diseases (GTDs) play a major role due to their high destructive effect and the high susceptibility of most commercial cultivars, being considered “the biotic stress of the century” for viticulture [5, 6]. GTDs are a complex of diseases caused by multiple coexisting fungal pathogens [7–9] that invade the lignified tissues, obstructing the xylem vessels, causing necrosis and producing hard and soft wood decay. This will lead to specific GTDs symptoms, that are similar across the different diseases [10, 11]. The destructive colonization of woody tissues is dependent on the presence of several virulence factors, including enzymes that degrade cell walls, toxins and a variety of cellular transporters [12]. Despite the incomplete understanding of the precise mode of action of these molecules, it is accepted that fungal toxins can inflict harm upon plant cells by interfering with enzymatic reactions and cellular transport, ultimately leading to the disruption of cell membranes [13]. Additional damage is linked to the host’s immune response to infection, particularly the formation of xylem vessel occlusions [13], which consequently impairs water and nutrient uptake. The development of GTDs symptoms may be even more complex than initially thought, due to the interaction of biotic and abiotic stresses [14]. Disease severity is influenced by the interaction between fungal life traits and host tolerance traits, as well as relationships with the whole microbiome [15]. Severity of symptoms varies between cultivars, but no resistance locus against GTDs has yet been identified [16, 17]. The fact that infected grapevines can remain externally asymptomatic for several years allowing diseases’ dissemination [18, 19], together with intensification of several management practices and the abolishment of chemical products [20] has led to a GTDs outbreak. GTDs are considered a major threat to winegrowers since no effective plant protection strategy is available [21, 22]. The incidence of this disease complex has reached alarming levels in several regions of Algeria, France, Germany, Italy, Spain, Portugal, and other countries [18], causing huge economic losses (up to one billion euros per year, estimated by the French Wine Institute), and threatening the viability of viticulture and the resulting wine industry [23, 24]. The management of GTDs has therefore become an enormous challenge and there is an urgent need to find and develop sustainable and effective protection strategies with lower environmental impact, while ensuring that production is both profitable and of high quality.

One of the most promising future strategies for protecting plants from GTDs is the development of pathogen-resistant cultivars [25, 26], not only to reduce pathogen management, but also to reduce the use and environmental impact of agrochemicals. Plant breeding of new cultivars carrying resistance genes can be developed through traditional genetic improvement, using directed crosses with closely related species that naturally carry resistance *loci*, although breeding programs are very prone to generate multiple unintended mutations and take a lot of resources and time; or through new breeding techniques (NBTs), including genome editing tools which are highly targeted and expeditious, and offer a more precise approach [25]. Despite regulatory challenges, the adoption of new resistant grapevine cultivars is gradually gaining traction, particularly in Europe [27], where recent regulations aim to promote the development of plants that are resilient to climate change and pests or diseases, and that exhibit higher yields or require reduced inputs of fertilizers and pesticides [28].

Knowledge of grapevine transcriptional responses to GTDs is crucial for understanding disease dynamics, as regulation of gene transcription is an essential step in an efficient host cell defence response, and for identifying key genes involved in tolerance mechanisms, which may indicate future breeding strategies. Nowadays, grapevine research is directed towards a better understanding of plant defence mechanisms and the characterization of the plant-pathogen interactions [17]. Several defence mechanisms have been described to prevent the penetration and colonization of pathogens in plants [22, 29]. Plant-pathogen interactions trigger the activation of signals that result in a rapid defence response against most plant pathogens [30–32]. Nevertheless, the huge system of host protection against aggressors is often ineffective to counteract disease’s development. Developments in genetics and technology have made it much easier to understand the molecular basis of grapevine-pathogen interactions and to study the molecular repertoires available for grapevine defence responses. Several resistance genes against specific pathogens have been functionally identified, although despite the various studies performed so far, a better knowledge of the complex molecular mechanisms underlying the grapevine defence responses against GTDs remains to be achieved [17]. Disease control involves prevention and mitigation, relying on basic viticultural practices and chemical and biological approaches. Nevertheless, the efficacy of these treatments will depend on their customization to specific fungal pathogens, together with environmental factors [9]. Therefore, there is a need to better understand the overall interactions that occur between grapevines and trunk pathogens, in order to mitigate their consequences and promote plant resistance. This will enable the development of new effective control strategies, environmentally friendly and with a minimal impact on human health.

Recent advances in omics technologies have been allowing a more comprehensive understanding of the molecular mechanisms underlying grapevine-pathogen interactions, enabling the identification of useful *loci* [33–36]. RNA-seq analysis takes advantage of the developments in next-generation sequencing (NGS) methods to enable a quantitative screening of distinct gene expression patterns in different plants at the transcriptome level, including in woody plants in response to phytopathogens. The analysis of differentially expressed genes (DEGs) and associated biological pathways provides valuable insights into the metabolic processes involved and helps to identify candidate genes with key regulatory functions, which is essential for the future of grapevine breeding.

This study aimed to understand the grapevine response mechanisms to GTDs, identifying key genes responsible for tolerance or susceptibility to this disease complex in grapevine. Since gene expression can change under different environments, the plants in our study were grown under natural field conditions, following a conventional farming system. The comprehensive analysis of the transcriptome was performed in symptomatic and asymptomatic plants to trunk diseases in two relevant cultivars in the Alentejo region, southern Portugal (in terms of surface area and yield), ‘Alicante Bouschet’ and ‘Trincadeira’, which reveal different susceptibility patterns to GTDs as referred in our previous study [37]. The results achieved will facilitate a more nuanced understanding of the resistance mechanism of grapevine against GTDs and enable future research on the application of genomic breeding systems to obtain resistant grapevine plants.

## Methods

### Study site and plant sampling

This study was conducted in a 17-year-old conventional cordon-pruned vineyard in the Alentejo region, southern Portugal (38°31′58.2″ N, 8°00′53.1″ W). The vineyard was subjected to regular phytosanitary treatments and managed under Integrated Pest Management rules, propagated on 1103P rootstock with a drip irrigation system. The field, influenced by the Mediterranean climate (with a mean annual temperature of 17.7 °C and annual rainfall of 627.7 mm in 2020 [38]), has a history of trunk diseases with many symptomatic grapevines.

In July 2020, 10 cm long spurs (one-year old and fully lignified) were randomly collected from three biological replicates of GTDs symptomatic and asymptomatic plants from cv. ‘Alicante Bouschet’ (highly susceptible to GTDs) and cv. ‘Trincadeira’ (slightly susceptible or tolerant to GTDs), totalling 12 samples. Symptomatic plants exhibited characteristic GTDs symptoms [37], while asymptomatic plants showed no visible symptoms on their wood or leaves. Available data on the susceptibility of these cultivars to GTDs are based on field observations by examination of foliar and/or wood symptoms [37, 39]. Samples were collected early in the morning to reduce abiotic stress and immediately preserved in liquid nitrogen until processing. In the laboratory, the rhytidome was removed and cortical scrapings from the cuttings were ground to powder, with each sample processed separately, using liquid nitrogen to aid the grinding, and stored at -80 °C until further analysis.

### RNA extraction and sequencing

Total RNA was extracted from each of the 12 collected samples using ca. 200 mg of the plant material powder, according to the method of Landi & Romanazzi [40], with some modifications. Briefly, the powder from each sample was placed in microtubes containing 1 mL of extraction buffer: 100 mM Tris-HCl (pH 8.0); 25 mM EDTA (pH 8.0); 2% (w/v) CTAB; 2% (v/v) β-mercaptoethanol; 2.5 M NaCl; 2% (w/v) soluble PVP-40, mixed by vortex and then were incubated at 65 °C for 45 min, with agitation. Lysates were transferred to QIAShredder spin columns (RNeasy Plant mini-kit, Qiagen) and centrifuged at 15 000 g for 2 min. Supernatants were transferred to new microtubes with an equal volume of chloroform-isoamyl alcohol (24:1), mixed, and centrifuged at 15 000 g for 10 min at 10 °C. The aqueous phase containing the total RNA was transferred to a new microtube and precipitated in 0.25 volume of 10 M LiCl, with the reaction left to proceed overnight at 4 °C. Samples were then centrifuged at 15 000 g for 30 min at 10 °C, the supernatants were removed, and the pellets were resuspended in 250 µL of 70% ethanol. Samples were centrifuged again at 15 000 g for 15 min at 10 °C, before being dried at room temperature inside the flow chamber. RNA was resuspended in 50 µL of ultrapure water and stored at -80 °C. RNA concentration was measured using Quawell Q9000 micro spectrophotometer (Quawell Technology, Beijing, China), and its purity was evaluated, based on 260/280 and 260/230 coefficients. RNA integrity was assessed by denaturing 1.5% agarose gel electrophoresis. Samples were then sent to a third-party facility, STAB VIDA, Lda. (Caparica, Portugal), for next-generation sequencing (NGS).

NGS project started with a quality control assessment of total RNA to ensure the integrity and quantity required for sequencing. RNA integrity was assessed using the RNA integrity number (RIN) determined with an Agilent 4200 TapeStation system (Agilent Technologies, Santa Clara, CA, USA), following the manufacturer’s guidelines. The RNA concentration of each sample was measured using a Qubit Fluorometer (Thermo Fisher Scientific, Waltham, MA, USA) to confirm sample suitability. The cDNA library construction was carried out using a Stranded mRNA Library Preparation Kit and the generated DNA fragments were sequenced in the lllumina Novaseq platform, using 150 bp paired-end sequencing reads. Analysis of the generated raw sequence data was performed using Qiagen CLC Genomics Workbench 12.0.3. Generated Fastq files were analysed using FastQC tool (v. 0.11.9) for quality control.

Bioinformatic analysis started with trimming of raw sequences to generate only high-quality data. For each original read, the regions of the sequence to be removed were determined independently considering each type of trimming operation: quality trimming based on quality scores (0.01 error probability); ambiguity trimming to trim off, for example, Ns stretches (2 nucleotides); and length trimming to remove small reads (minimum 30 nucleotides). High-quality sequencing reads were mapped against the grapevine reference genome, GCA_000003745.2 *Vitis vinifera* 12X [41], using the following parameters: length fraction greater than or equal to 0.8, meaning that at least 80% of the alignment must match the reference sequence before the read is included in the mapping, and similarity fraction also greater than or equal to 0.8, meaning that the identity should be at least 80% for the read to be included in the mapping. The result of the mapping was used to determine gene expression levels based on transcripts per million (TPM), which is a variation of the reads per kilobase of exon model per million mapped reads (RPKM) method, calculated by dividing the total number of reads for a gene by the total number of mapped reads of the transcriptome (in millions) times the length (in kb) of that gene [42].

### Differential gene expression and functional enrichment analysis

A generalized linear model approach influenced by the multi-factorial EdgeR method [43] was used for differential expression analysis. This model corrects for differences in library size between samples and the effects of confounding factors. Genes were filtered out by a false discovery rate (FDR) of ≤ 0.05 and a fold change of ≥ 2.0 or ≤ -2.0 [44, 45], and were considered differentially expressed with a *q*-value adjusted, using the Bonferroni correction method, > 0 and < 0.05.

The list of DEGs from each comparison was analysed using the enricher function from the clusterProfiler v4.2.2 package [46] to identify significantly enriched functional categories based on Gene Ontology (GO) terms. The enricher function applies a hypergeometric test, and the resulting gene ratio is calculated as the ratio of the number of DEGs to the total number of genes in specific GO terms. Functional enrichment analysis was performed using Fisher’s exact test to compare each list of DEGs with the list of total non-redundant transcripts housed in the grapevine 12X gene predictions [47]. Significantly enriched categories were selected based on an adjusted *p*-value ≤ 0.05, using the Benjamini–Hochberg correction for multiple testing. Analyses were performed for the three ontology levels: molecular function, cellular process and biological process.

### Real time quantitative PCR (qPCR) validation analysis

Validation of the RNA-seq data was performed using 16 target genes: *PR2* Glucan endo-1,3-β-D-glucosidase (VIT_00035013001); *LOX* Linoleate 9 S-lipoxygenase (VIT_00000083001); *STS1* Stilbene synthase 1 (VIT_00010561001); *HT5* Hexose transporter (VIT_00017937001); *cwINV* β-fructofuranosidase, cell wall apoplastic invertase (VIT_00016869001); *GIN2* Acid β-fructofuranosidase, vacuolar invertase (VIT_00001272001); *PER42* Peroxidase 42 (VIT_00012727001); *PR1* Pathogenesis-related protein 1 (VIT_00037005001); *PR4* Pathogenesis-related protein 4 (VIT_00036279001); *TLP8* Thaumatin family protein (VIT_00019840001); *TLP3* Thaumatin family protein (VIT_00019835001); *MAPKKK17* Mitogen-activated protein kinase kinase kinase 17 (VIT_00030452001); *HSP101* Heat Shock Protein 101 (VIT_00007880001); *bHLH94* Transcription factor (VIT_00000012001); *SAUR71* Auxin-responsive protein (VIT_00036807001); and *MYB61* Transcription factor (VIT_00019410001) [16, 48–51]. Primers of *bHLH94, SAUR71* and *MYB61* were designed using Primer3 software version 0.4.0, based on the specific sequence of *V. vinifera* available in the NCBI GenBank. Additional information on the selected primers can be found in the Supplementary Table S1.

Total RNA (1000 ng) from each of the 12 collected samples was reverse transcribed in 20 µL reactions, using the Maxima^®^ First Strand cDNA Synthesis Kit for qPCR (Thermo Fisher Scientific, Waltham, MA, USA) according to the manufacturer’s instructions. qPCRs were carried out using the LineGene9600 Plus system (BIOER, Hangzhou, China) and were performed using 5 µL of first-strand cDNA (previously diluted 1:10, with a final concentration of 5 ng/µL), 9 µL of 2x NZY qPCR Green Master Mix (Nzytech, Lisbon, Portugal), 0.55 µM of each primer, for a total volume of 18 µL. Three technical replicates were considered for each of the three biological replicates and no template controls were included to assess contaminations. Cycling conditions included an initial holding denaturation stage at 95 °C for 20 s, followed by 40 amplification cycles of 15 s at 95 °C and 20 s at 60 °C. A melting stage to test PCR specificity was added as a final step comprising a single cycle at 95 °C/15 seconds followed by 60 °C/1 min, and a ramp-up 0.2 °C/second to 95 °C for 15 s with acquired fluorescence. qPCR efficiencies were calculated through the equation $\:E=\left(10\left(-1/slope\right)-1\right)\times\:100$, as well as slope and linearity (coefficient of determination, R^2^), using 4-point standard curves from 4-fold dilution series (1:10, 1:40, 1:160, and 1:640) of pooled cDNA (Supplementary Table S1).

*GAPDH* Glyceraldehyde-3-phosphate dehydrogenase (VIT_00007521001), *PEP* Phosphoenolpyruvate carboxylase (VIT_00020705001) and *UBC* Ubiquitin conjugating enzyme (VIT_00027045001) [16, 52] were the most stable genes tested using the geNorm software 3.0 [53] and were, therefore, chosen as endogenous control genes for target normalization. Cycle threshold values were regressed on the Log of the previously constructed template cDNA curve. The normalized arbitrary units of the target genes for each sample were then calculated using the normalization factors obtained for the reference genes.

To determine if the gene expression values were significantly different between the experimental groups, a student’s t-test was executed using IBM SPSS Statistics software (IBM SPSS Statistics for Windows, version 24.0. Armonk, NY, USA: IBM Corp.) with an established significance level of *p* < 0.05. Equal variances of the samples were checked with Levene’s Test for Equality of Variances with values lower than 0.05 not considered as equal variances and another Independent Samples Test was performed assuming no equal variances (Supplementary Table S2). For each gene, the Log_2_ fold change of qPCR was compared with the RNA-seq, and Pearson correlation coefficients and associated *p*-values were also estimated. To measure the level of agreement between these two methods, the concordance correlation coefficient [54] was estimated using the Log_2_ fold change values per candidate gene. Two experimental groups were considered, namely ‘Trincadeira’ vs. ‘Alicante Bouschet’ (cultivar factor) and symptomatic vs. asymptomatic plants (symptomatology factor).

## Results

### Transcriptome sequencing of grapevine plants: mapping statistics and annotation

RNA-seq analysis was performed to access the expression profiles of GTDs symptomatic and asymptomatic plants of two red cultivars with different levels of GTDs susceptibility, ‘Alicante Bouschet’ and ‘Trincadeira’, to understand if there is any molecular mechanism that leads to the expression of trunk diseases symptoms.

More than 694 million reads were initially obtained with an average of over 57 million raw reads per sample with an average read length of 150 bp (Supplementary Table S3). All samples shared an average associated Phred quality score over 20. The expression levels for all samples were accessed through the mapping of the high-quality reads of each sample, where 90.89% to 94.78% of the total fragments were mapped against the *Vitis vinifera* 12X reference genome, which is in line with other grapevine transcriptome studies [35, 55].

In addition, Principal Component Analysis (PCA) revealed a clear clustering pattern and intrinsic biological variance between all sample replicates, confirming the good reproducibility of the biological replicates and the high-quality of sequencing data. Principal component 1 explained 35.20% of the variation and principal component 2 accounted for 16.10%, which combined analysis of variance revealed 51.30% among different samples (Supplementary Figure S1).

### Differential gene expression analysis

RNA-seq data revealed the expression of 26 346 grapevine genes across all samples. Differential gene analysis was performed by multiple comparisons in six comparison groups: ‘Trincadeira’ vs. ‘Alicante Bouschet’ (T vs. AB); symptomatic vs. asymptomatic plants (symp. vs. asymp.); symptomatic plants from cv. ‘Alicante Bouschet’ vs. symptomatic plants from cv. ‘Trincadeira’ (AB symp. vs. T symp.); asymptomatic plants from cv. ‘Alicante Bouschet’ vs. asymptomatic plants from cv. ‘Trincadeira’ (AB asymp. vs. T asymp.); symptomatic vs. asymptomatic plants from cv. ‘Alicante Bouschet’ (AB symp. vs. AB asymp.); and symptomatic vs. asymptomatic plants from cv. ‘Trincadeira’ (T symp. vs. T asymp.), resulting in a total of 1 598 genes identified as differentially expressed between cultivars (622 up-regulated in cv. ‘Trincadeira’ and 976 in cv. ‘Alicante Bouschet’) and 64 genes between symptomatic and asymptomatic plants (59 up-regulated in symptomatic and 5 in asymptomatic plants) (Table 1). Major differences were found between both asymptomatic groups, with a total of 1 628 DEGs, and the fewest differences were found between groups with different symptomatology, regardless of the cultivar (Table 1). The full detailed lists of DEGs for the six comparison groups can be found in Supplementary Table S4. Volcano plots were also used to visualize the pattern of DEGs in the different comparison groups (Supplementary Figure S2). Hierarchical clustering and the heat map of differential gene expression levels for each sample were also performed and are presented in Supplementary Figure S3.

**Table 1:** Differentially expressed genes (DEGs) identified in different comparison groups of GTDs symptomatic (symp.) and asymptomatic (asymp.) plants from cv. ‘Alicante Bouschet’ (AB) and ‘trincadeira’ (T)

Comparison Groups	Total DEGs	Up-regulated	Down-regulated
T vs. AB	1 598	622	976
symp. vs. asymp.	64	59	5
AB symp. vs. T symp.	1 438	790	648
AB asymp. vs. T asymp.	1 628	1 070	558
AB symp. vs. AB asymp.	185	91	94
T symp. vs. T asymp.	375	277	98

**Fig. 1:**
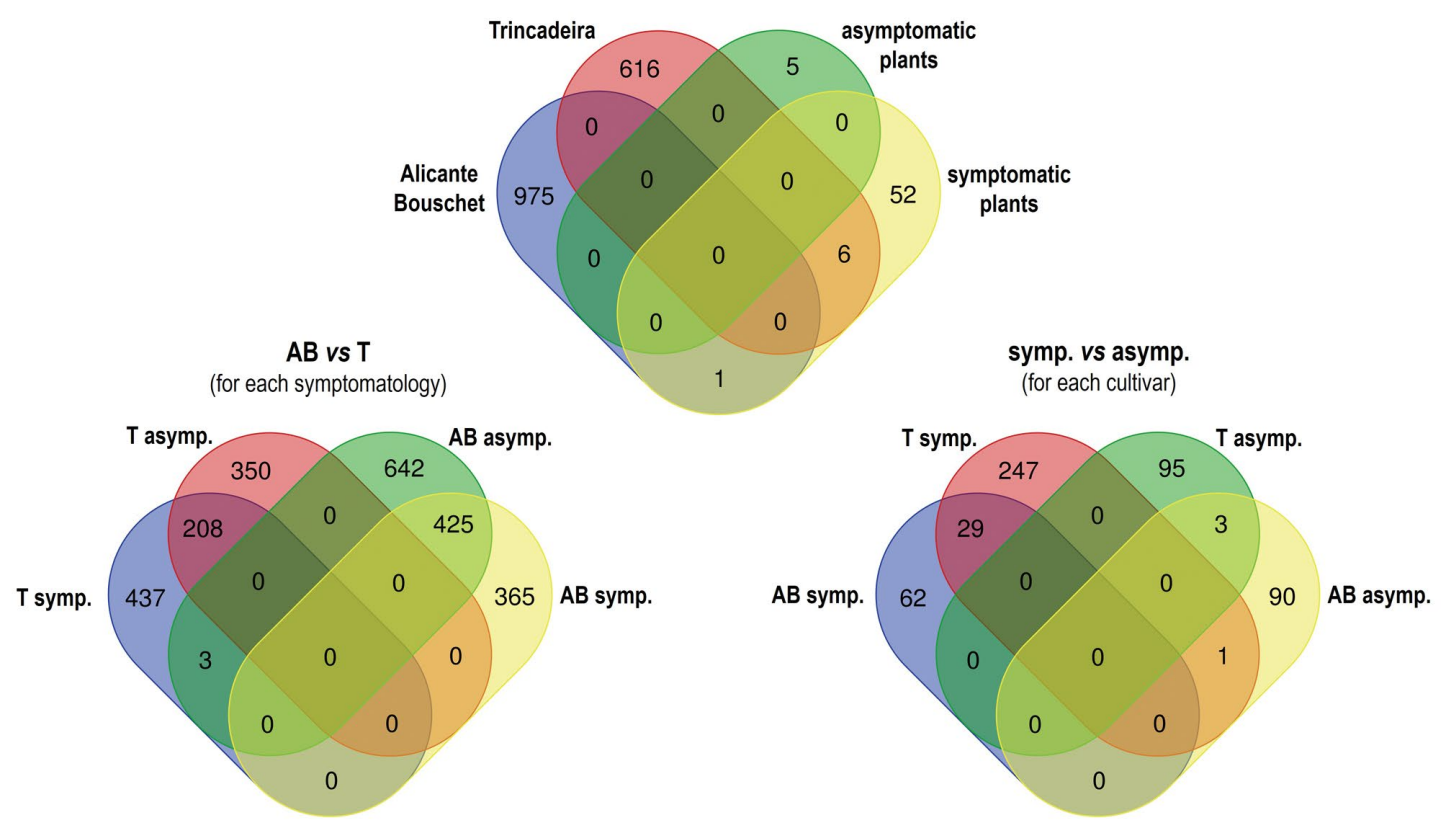
Venn diagrams with the common differentially expressed genes (DEGs) between GTDs symptomatic (symp.) and asymptomatic (asymp.) plants from cv. ‘Alicante Bouschet’ (AB) and ‘Trincadeira’ (T)

The Venn diagrams illustrate that there are some common genes between the groups, one between ‘Alicante Bouschet’ and symptomatic plants (VIT_00031801001 - peroxidase 10) and six between ‘Trincadeira’ and symptomatic plants (VIT_00018906001 - salicylate carboxymethyltransferase, VIT_00016932001 - cryptdin protein, VIT_00017634001 - inositol 3-alpha-galactosyltransferase, VIT_00012936001 - trypsin and protease inhibitor, VIT_00036051001 - NDR1/HIN1-like protein and VIT_00009849001 - cytochrome P450 71B21-related / 5-OH-xanthotoxin synthase). When comparing symptomatic plants of both cultivars, ‘Trincadeira’ has a lower number of DEGs (Table 1), although it has a higher number of DEGs exclusive to symptomatic plants (Fig. 1). On the other hand, ‘Alicante Bouschet’ has a greater number of genes exclusive to asymptomatic plants (642 genes). The same is observed when we analyse symptomatic and asymptomatic plants of the same cultivar. There are three common DEGs in asymptomatic plants and 29 in symptomatic ones, with ‘Trincadeira’ having a much higher number of up-regulated genes (Fig. 1).

### Validation analysis

**Fig. 2:**
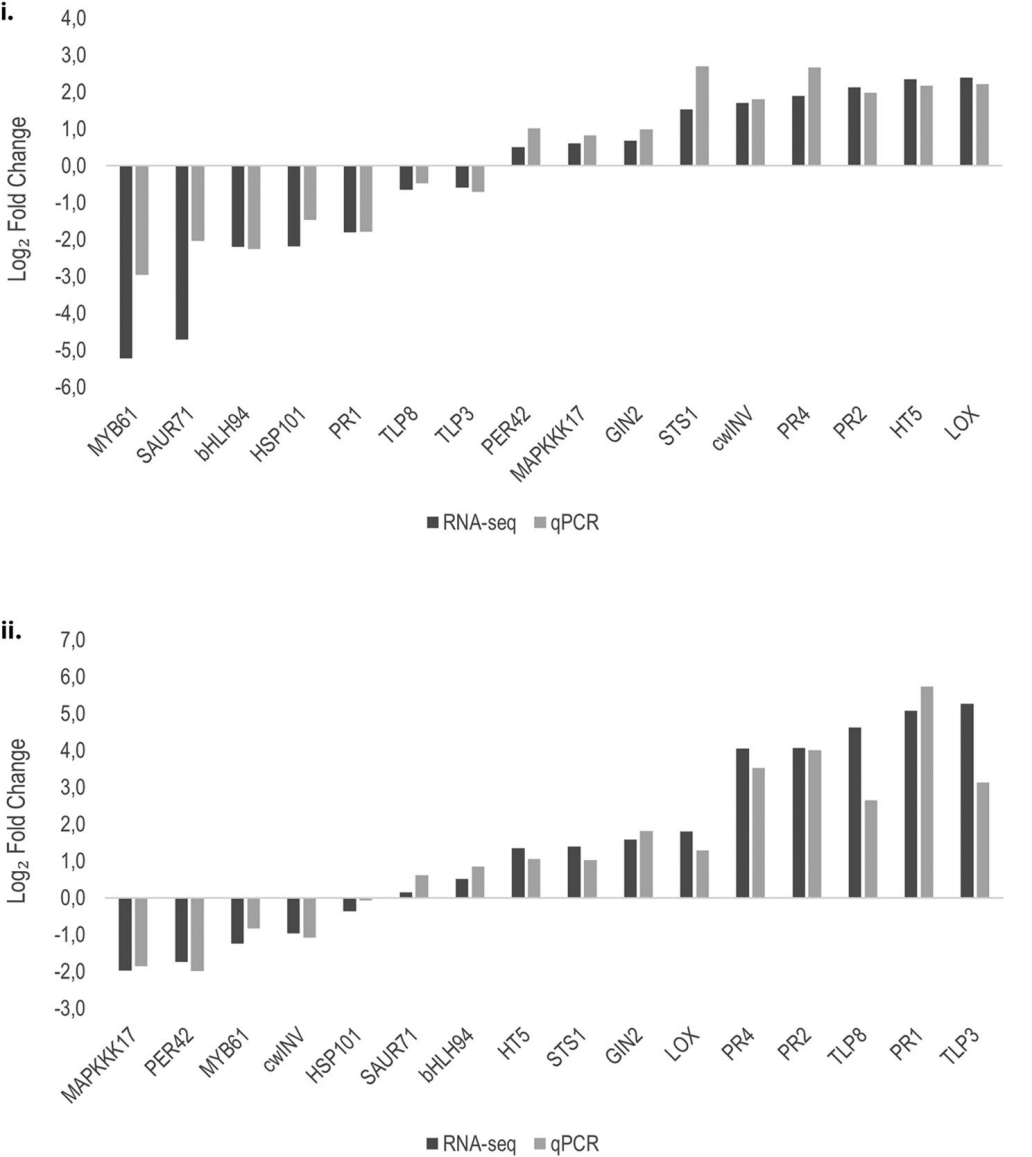
Gene expression comparison of 16 genes with RNA-seq and qPCR of GTDs symptomatic (symp.) and asymptomatic (asymp.) plants from cv. ‘Alicante Bouschet’ (AB) and ‘Trincadeira’ (T), considering only two comparison groups **(i)** T vs. AB and **(ii)** symp. vs. asymp. Positive values indicate overexpression in ‘Trincadeira’ and symptomatic plants; and negative values overexpression in ‘Alicante Bouschet’ and asymptomatic plants. PR1 Pathogenesis-related protein 1; PR2 Glucan endo-1,3-β-D-glucosidase; PR4 Pathogenesis-related protein 4; TLP3 and TLP8 Thaumatin family; bHLH94 and MYB61 Transcription factors; MAPKKK17 Mitogen-activated protein kinase kinase kinase 17; PER42 Peroxidase 42; LOX Linoleate 9 S-lipoxygenase; SAUR71 Auxin-responsive protein; STS1 Stilbene synthase 1; HSP101 Heat Shock Protein 101; HT5 Hexose transporter; GIN2 Acid β-fructofuranosidase, vacuolar invertase; cwINV β-fructofuranosidase, cell wall apoplastic invertase. The significance of the correlation is *p* < 0.001 for all target genes

To validate the accuracy and reliability of our transcriptome data, 16 target genes with different expression levels were selected for qPCR analysis (Supplementary Figure S4). For each gene, the Log_2_ fold change of qPCR was compared with the RNA-seq (Fig. 2), and Pearson correlation coefficients ranged from 0.897 (*SAUR71*) to 0.998 (*PR2*) (Supplementary Figure S5). The concordance correlation coefficient between these two methods was determined, and the obtained values were 0.843 and 0.879 for the cultivar and symptomatology factors, respectively. The results demonstrate a substantial strength of agreement between RNA-seq and qPCR analysis, indicating that the RNA-seq data reflects the real expression patterns of the grapevine genes.

### Functional enrichment analysis of differentially expressed genes

**Fig. 3:**
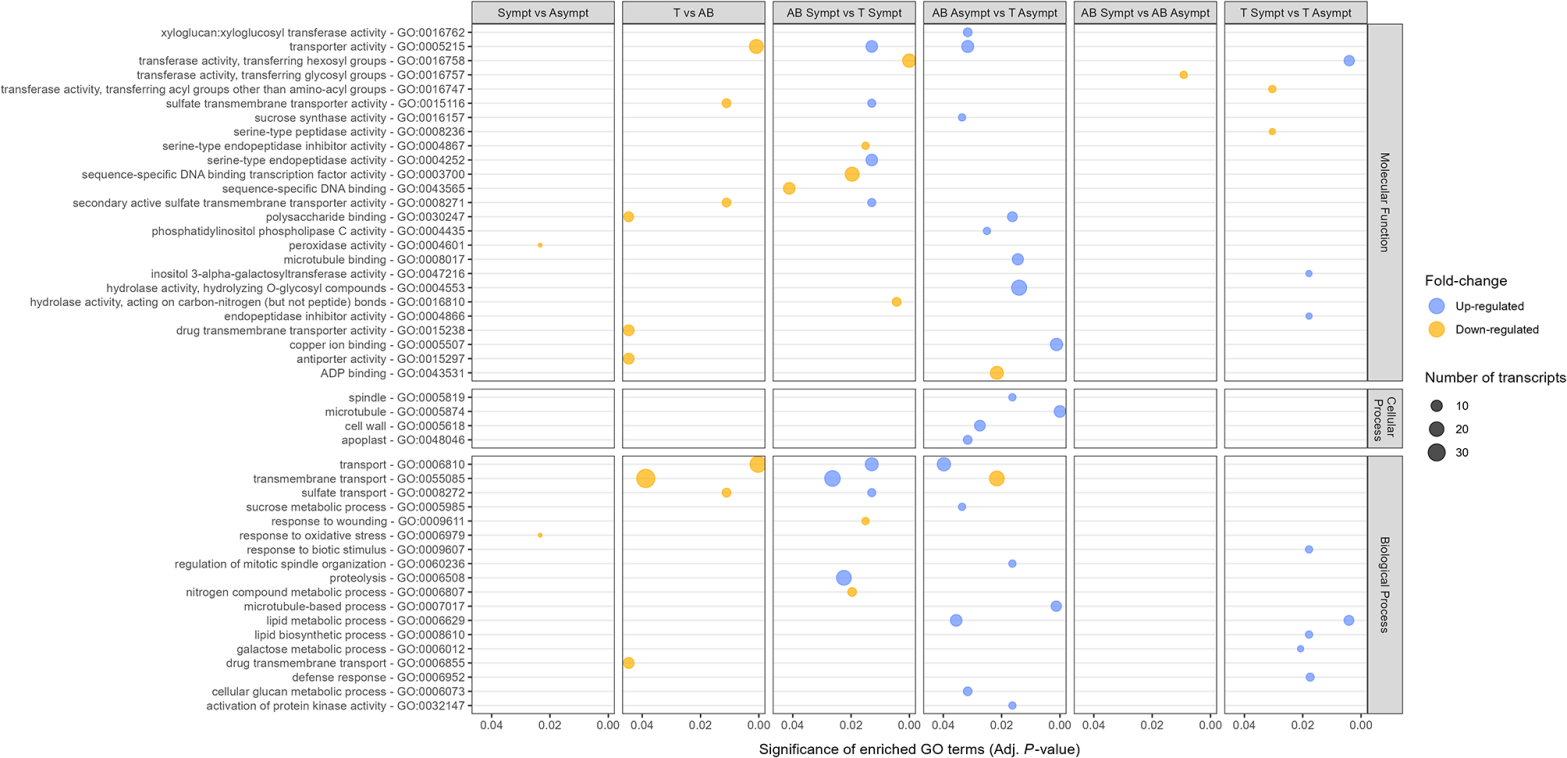
Significantly enriched GO terms of DEGs in response to GTDs infection (p-adjust ≤ 0.05) in six comparison groups: symptomatic vs. asymptomatic plants (symp. vs. asymp.); ‘Trincadeira’ vs. ‘Alicante Bouschet’ (T vs. AB); symptomatic plants from cv. ‘Alicante Bouschet’ vs. symptomatic plants from cv. ‘Trincadeira’ (AB symp. vs. T symp.); asymptomatic plants from cv. ‘Alicante Bouschet’ vs. asymptomatic plants from cv. ‘Trincadeira’ (AB asymp. vs. T asymp.); symptomatic vs. asymptomatic plants from cv. ‘Alicante Bouschet’ (AB symp. vs. AB asymp.); and symptomatic vs. asymptomatic plants from cv. ‘Trincadeira’ (T symp. vs. T asymp.). Blue and yellow points represent up- and down-regulated genes associated to specific pathways, respectively. The size of each point indicates the number of genes involved in each functional subcategory. Full dataset in Supplementary Table S5

Functional enrichment analysis of up- and down-regulated genes was performed (Fig. 3; Supplementary Table S5) based on functional categories [47]. A separate functional analysis is presented for the six comparisons: T vs. AB; symp. vs. asymp.; AB symp. vs. T symp.; AB asymp. vs. T asymp.; AB symp. vs. AB asymp.; and T symp. vs. T asymp. Overall, the GO enrichment analysis indicated that GTDs infection had a greater influence on DEGs belonging to the categories of molecular function, followed by biological process, and finally to cellular process.

In the symp. vs. asymp. comparison group, only two enriched GO terms related to peroxidase activity and response to oxidative stress were identified. In both, the single gene involved is *PER42*, up-regulated in asymptomatic plants. When comparing both cultivars (T vs. AB), regardless of the symptomatology, 10 enriched GO terms were identified, including six molecular functions (transporter activity, sulfate transmembrane transporter activity, secondary active sulfate transmembrane transporter activity, polysaccharide binding, drug transmembrane transporter activity, and antiporter activity) and four biological processes (transport, transmembrane transport, sulfate transport, and drug transmembrane transport), all of them related to transport and markedly enriched in ‘Alicante Bouschet’, the susceptible cultivar. These DEGs included those encoding multidrug and toxic compound extrusion (*MATE*) transporters, glycosyltransferases, wall-associated receptor kinases (*WAK*) and other protein kinases, sulfate transporters, nitrate-peptide transporters, ATP-binding cassette (*ABC*) transporters, sugar transporters, nucleobase-ascorbate transporters, voltage and ligand gated potassium channel, chloride channel and mechanosensitive ion channel proteins, Ca^2+^:H^+^ antiporter, auxin efflux carrier family protein, aquaporins, among others.

In the AB symp. vs. T symp. comparison group, there was an enrichment of 15 GO terms, eight of them up-regulated, that include genes involved in transport, which largely overlap with those in the previous comparison group, as well as genes involved in fundamental processes of primary metabolism for recycling and regulating cellular protein content (serine-type endopeptidase activity and proteolysis). The remaining seven enriched GO terms include genes up-regulated in T symp. involved in primary metabolism (transferase activity - transferring hexosyl groups, hydrolase activity - acting on carbon-nitrogen bonds, and nitrogen compound metabolic process), regulation of gene expression (serine-type endopeptidase inhibitor activity, sequence-specific DNA binding transcription factor activity, and sequence-specific DNA binding), and stress response (response to wounding). These included genes belonging to the β-cyano-L-alanine hydratases/nitrilases, UDP-glycosyltransferases, WRKY, heat shock, AP2/ethylene-responsive transcription factor and other families, and potato inhibitor I family. The presence of various potato type I and II proteinase inhibitors has been verified in response to pathogens, with an altered regulation of different plant physiological processes (e.g. response to dehydration, programmed cell death, plant growth, trichome density, and branching) [56].

AB asymp. vs. T asymp. was the most enriched group with 19 up-regulated GO terms. The highly expressed are hydrolase activity - hydrolyzing O-glycosyl compounds (22 genes), copper ion binding (13 genes), transport (16 genes), and transporter activity (12 genes). The down-regulated genes are involved in transmembrane transport (21 genes) and ADP binding (15 genes). DEGs of phosphatidylinositol phospholipase C, hydrolase, xyloglucan: xyloglucosyl transferase, activation of protein kinase and sucrose synthase activities, sucrose metabolic and cellular glucan metabolic processes, regulation of mitotic spindle organization, apoplast, cell wall, microtubules, spindle, copper ion and microtubule binding, and microtubule-based process were exclusively up-regulated in asymptomatic ‘Alicante Bouschet’ plants.

In contrast, the AB symp. vs. AB asymp. comparison group was the least enriched group regarding GO terms, with only one enriched molecular function related to transferase activity - transferring glycosyl groups, including two galactinol synthase and one inositol 3-alpha-galactosyltransferase genes up-regulated in AB asymp. These genes are involved in the biosynthesis of raffinose oligosaccharides, which has been widely described to perform several functions in plants, including carbohydrate transport and protection against biotic and abiotic stresses [57]. The accumulation of these oligosaccharides can help to reduce oxidative damage by scavenging reactive oxygen species (ROS) produced during stress. Their role in the accumulation of protective sugars may help to limit pathogen spread and increase the overall resilience of the plant under stressful conditions, which may help ‘Alicante Bouchet’ plants to avoid showing GTDs symptoms [58]. No functional category was enriched in symptomatic plants of ‘Alicante Bouschet’.

Finally, two molecular functions related to primary metabolism (transferase activity - transferring acyl groups other than amino-acyl groups and serine-type peptidase activity) were up-regulated in T asymp. when comparing symptomatic and asymptomatic plants of ‘Trincadeira’ (T symp. vs. T asymp.). On the other hand, three molecular functions and five biological processes were up-regulated in T symp. These include genes involved in primary and secondary metabolism (transferase activity - transferring hexosyl groups, inositol 3-alpha-galactosyltransferase activity, galactose metabolic process, lipid metabolic process, and lipid biosynthetic process), regulation of gene expression (endopeptidase inhibitor activity), and stress response (response to biotic stimulus and defence response).

## Discussion

### The number of DEGs is mainly impacted by grapevine cultivar

Plant health is strongly linked to the intrinsic immunity of the plant, which depends on the induction of defence responses. Transcriptional changes in both plant resistant and susceptible response to pathogens help to exploit key genes involved in the activation of antipathogenic responses for the development of a molecular toolbox that can estimate the tolerance or resistance to diseases [59, 60].

In the study here presented, differences in gene expression were analysed by comparing the transcriptomic profile of GTDs symptomatic and asymptomatic plants of cv. ‘Alicante Bouschet’ (highly susceptible to GTDs) and cv. ‘Trincadeira’ (slightly susceptible or tolerant to GTDs). As expected, our results indicated that GTDs infection stimulated a wide range of responses in the host and demonstrated that defensive strategies were more effective in ‘Trincadeira’. In contrast, ‘Alicante Bouschet’ did not elicit such defensive responses and displayed an enrichment of functional categories predominantly associated with transport, which may contribute to the heightened susceptibility of this cultivar. Although samples selected for transcriptomic profiling were collected after the onset of GTDs symptoms, the degree of senescence observed is likely to reflect the different susceptibility of the cultivars to the disease. More susceptible cultivars might exhibit more pronounced senescence, which could influence the observed gene expression patterns. Notably, differences in cultivar susceptibility to Esca were associated to variations in vascular occlusion formation, which is linked to premature senescence, and may serve as an indicator of plant resistance [61]. This further supports that the differential responses result from both disease impact and inherent cultivar-specific defense mechanisms.

PCA indicated that most of the observed variation in gene expression was a consequence of the cultivar, with the largest differences were observed between the two asymptomatic groups (AB asymp. vs. T asymp.). This is explained by the genetic divergence between the two *V. vinifera* cultivars. A complementary microsatellite study, carried out by our team, confirmed that the selected plants belonged to the expected cultivar (data not shown).

### Transport-related genes are suggested to play an important role in the susceptibility of ‘Alicante Bouschet’ to trunk diseases

Transcriptomic data revealed that genes up-regulated in ‘Alicante Bouschet’, the most susceptible cultivar, when comparing with ‘Trincadeira’, were enriched in functional categories mainly involved in transport, especially in symptomatic plants (Fig. 3; Supplementary Table S5). Transporter proteins facilitate the movement of various substances across cell membranes, including a wide repertoire of secondary metabolites, toxins, metal ions, and other small compounds [62, 63], which may influence plant-pathogen interactions. In the case of ‘Alicante Bouschet’, the up-regulation of transport-related genes suggests a multifaceted involvement in the disease process, possibly promoting pathogen dissemination or enhancing the movement of toxic metabolites within the plant.

Secondary metabolites play a role in plant defence against pathogens, including in grapevine [63, 64]. However, not all the secondary metabolites present in plants are produced by them; many are produced by pathogens, including phenolic phytotoxins, growth-related phytohormones and toxic effectors [15], which may play a role in virulence in plant-pathogen interactions and may be linked to symptom development [65]. This can explain the high amount of up-regulated genes encoding transporter proteins found in ‘Alicante Bouschet’ compared to ‘Trincadeira’, especially in symptomatic plants. Symptomatic plants from a susceptible cultivar would be expected to show higher levels of secondary metabolites, enhancing symptom development. The accumulation of phytotoxic metabolites and cell-wall-degrading enzymes in the lumens of xylem vessel restricts water transport, leading to foliar symptoms [66, 67], and induces the deposition of tyloses as a defensive response [68]. The role of phytotoxic metabolites in the virulence of trunk pathogens has been the subject of considerable research [12, 69, 70].

A prominent group of transporters overexpressed in ‘Alicante Bouschet’ plants is the ABC superfamily. These transporters may contribute to detoxification and defence, and might inadvertently aid pathogen survival by mediating toxin efflux or redistributing compounds that favour infection [63, 71]. An up-regulation of ABC transporters was already identified during grapevine infection with *Lasiodiplodia theobromae* [72].

Some MATE genes were overexpressed in ‘Alicante Bouschet’ plants, both in the comparison between cultivars and between symptomatic plants of the two cultivars. The MATE protein family is highly abundant in plants and plays a pivotal role in regulating various growth and developmental processes, including the transport of secondary metabolites, toxic compounds and heavy metals; the regulation of disease resistance; and the modulation of plant hormone signalling pathways [73]. Their function in disease resistance is complex: while they can contribute to detoxification, excessive activity may lead to the depletion of defence-related secondary metabolites. The overexpression of MATE genes in ‘Alicante Bouschet’ suggests a potential dysregulation of secondary metabolite transport, possibly interfering with an effective defence response and increasing pathogen fitness, as already verified in other species [74, 75].

Pathogen infection is known to trigger innate immunity via pathogen-associated molecular patterns-triggered immunity (PTI) through extracellular pattern-recognition receptors (PRRs) located on the plasma membrane of the host cells [29]. WAKs are receptor-like kinases that encode PRRs capable of recognizing both cell wall-derived pectins and pectin fragments resulting from pathogen activity, thereby activating defence responses [76]. These genes were up-regulated in ‘Alicante Bouschet’ plants, indicating activation of PTI, leading to the ROS production and epigenetic modifications that regulate the expression of several defence-related genes. Although ROS are essential for signalling and defence, their excessive accumulation may lead to oxidative stress, which damages cell structures, including membranes and cell walls [77]. This oxidative damage results in a grapevine weakening, rendering it more susceptible to invasion by trunk pathogens.

Moreover, genes associated with water and nutrient transport may contribute to the systemic dissemination of trunk pathogens or their toxic metabolites within the grapevine, which rely on the vascular system to move through the plant, potentially leading to more extensive colonization and damage. Aquaporins are regarded as the primary molecular gateway for water entry into cells, and they regulate a multitude of physiological processes [78]. Their expression has been demonstrated to be altered in response to a range of environmental stresses, including drought and nutrient deficiency or toxicity [79]. The up-regulation of several aquaporins in ‘Alicante Bouschet’ may reflect an altered water transport system in response to pathogen-induced xylem occlusion, as already observed in apoplectic grapevines [80].

Furthermore, our findings revealed a notable overexpression of the transcription factor *MYB61* and *bHLH94* in ‘Alicante Bouschet’ when compared to ‘Trincadeira’. MYB transcription factor family plays a pivotal role in the regulation of genes that control the formation of secondary cell walls, which is of paramount importance for providing structural support and facilitating water and nutrient transport in plants, particularly in vascular tissues such as xylem [81, 82]. The bHLH transcription factor family has been demonstrated to exert significant influence over the regulation of genes associated with iron uptake, transport, and homeostasis [83].

To suppress the PTI response, pathogens have evolved specialized secretory systems that facilitate the transport of effectors into plant cells and enable efficient host colonization [84]. To overcome these pathogen effectors, plant R genes recognize these toxic effectors and activate the second defence line, known as ETI [31, 60, 85]. A comparative analysis of the symptomatic plants of both cultivars and asymptomatic plants, separately, revealed that several other genes coding for kinase receptors, including leucine-rich repeat receptor kinases (LRR-RKs) and nucleotide binding site (NBS)-leucine-rich repeat (LRR) receptors (NBS-LRR), were up-regulated in ‘Trincadeira’, mainly in asymptomatic plants. The involvement of several putative NBS-LRR and other R genes in grapevine response to different pathogens were already pointed [35, 86–88]. Therefore, we hypothesize that these receptors may be a significant factor in the restriction of infection by GTDs in the tolerant cultivar ‘Trincadeira’.

Unlike ‘Alicante Bouschet’, which appears to over-activate transporters in response to infection, ‘Trincadeira’ maintains a more balanced regulation of these genes, potentially preventing excessive movement of pathogen-facilitating compounds. This suggests that ‘Trincadeira’ benefits from a more coordinated defence response, that might contribute to its lower susceptibility to trunk diseases. Overall, the data indicates that ‘Alicante Bouschet’ activates the first line of defence in response to GTDs, but the responses are not enough to restrict fungal growth or to mitigate disease progression. In addition, the overexpression of transport-related genes plays an essential role in its susceptibility to trunk pathogens through several mechanisms: facilitating pathogen or toxin movement within the plant, particularly via ABC and MATE transporters; leading to oxidative stress due to imbalanced ROS transport via aquaporins; and interfering with defence signalling by modulating secondary metabolite distribution, potentially weakening the plant’s resistance mechanisms. Conversely, ‘Trincadeira’ activates the second line of defence, which is expressed as receptors that restrict fungal infection more effectively and may potentially benefit from a more regulated gene expression, thereby maintaining superior overall resistance to these diseases.

### GTDs symptoms prompt a response from the ‘Trincadeira’ transcriptome, putatively regulated by secondary and hormonal metabolism biosynthesis

The number of DEGs increased significantly in the presence of GTDs symptoms in cv. ‘Trincadeira’, in contrast with ‘Alicante Bouschet’ (Table 1; Supplementary Table S4). Although symptomatic ‘Trincadeira’ plants have fewer DEGs compared to those of ‘Alicante Bouschet’, the more tolerant cultivar has a higher number of DEGs exclusively in symptomatic plants (Fig. 1). GO analysis revealed that the DEGs that were up-regulated in symptomatic plants of cv. ‘Trincadeira’ were enriched in multiple pathways associated with primary and secondary metabolism, regulation of gene expression and stress response. In contrast, these pathways were not enriched in the susceptible cultivar (Fig. 3; Supplementary Table S5). Our results also revealed that symptomatic ‘Trincadeira’ plants presented a higher number of activated categories compared to the asymptomatic ones, including response to biotic stimulus and defence response (Fig. 3). The genes belonging to these functional categories include three PR genes and a Sect. 61 protein translocation complex (β subunit), also known as Mildew Locus O 13 (*MLO13*) - susceptibility gene [89, 90]. Pathogens may exploit *MLO* genes to regulate the transport of metabolites during papillae formation, forcing the plant to form only non-effective papillae [91]. Some *MLO* genes were up-regulated upon *Erysiphe necator* inoculation and may act as susceptibility genes [35], as their knockdown resulted in a reduction in powdery mildew severity in grapevines [89]. Although *MLO13* knockdown did not reduce powdery mildew severity [89], its overexpression in symptomatic ‘Trincadeira’ plants suggests a potential role in susceptibility to GTDs.

PR-encoding genes are present at low levels even in healthy plants. However, it is known that these genes have been induced in a wide range of plant species in response to pathogens, acting as the first line of plant defence [22, 92]. These proteins play an important role in early defence events, with direct antimicrobial properties (e.g., osmotins and thaumatins) through hydrolytic activities on the pathogen cell wall (e.g., β-1,3-glucanases and chitinases), and/or indirectly leading to the production of elicitors that trigger additional defence responses against phytopathogens [93]. Several PR-encoding genes have been observed to be differentially influenced in various grapevine cultivars in response to a wide range of pathogens [16, 33, 94]. In our study, both cultivars display the synthesis of PR proteins in response to fungal pathogen infection, which suggests that GTDs are perceived by the host. *PR4*, *TLP8* and *TLP3* were up-regulated in ‘Alicante Bouschet’ symptomatic plants, when compared to asymptomatic ones, and all of these genes, along with *PR2*, were overexpressed in symptomatic ‘Trincadeira’ plants. Moreover, three additional PR-encoding genes related to defence response and response to biotic stimulus were up-regulated in the T symp. plants. The induction of these genes may result in the hydrolysis of fungal cell wall components and the release of β-1,3 glucans and chitin fragments, which are effective elicitors that amplify plant defence responses. Nevertheless, the positive regulation of the expression of some PR genes, grapevine plants expressed symptoms, indicating that their induction in our plant pathosystem was not enough to prevent the expression of symptoms and limit infection, as also reported by Magnin-Robert et al. [80].

Secondary metabolism is markedly activated in grapevine following infection by a wide range of pathogens [95]. The present study also reveals an enrichment in the secondary metabolism biosynthesis in cv. ‘Trincadeira’ in the presence of GTDs symptoms, mostly associated with stilbenoid, flavonoid and phenylpropanoid pathways. A significant increase in the expression of stilbene synthase (*STS1*), which is responsible for the biosynthesis of resveratrol and stilbenoid compounds, a class of secondary metabolites with antimicrobial properties, has been reported in response to Esca and Eutypa dieback diseases [16, 80, 96]. Several studies highlight the accumulation of stilbenes (classified as phytoalexins) in diseased grapevine wood, which possess antifungal properties and are implicated in plant defence [97, 98]. Genetic manipulation of phytoalexins has been explored to enhance plant disease resistance, particularly through the overexpression of *STS1*, leading to increased stilbene production [31]. Resveratrol has been identified as a key compound in grapevine defence against trunk pathogens, although its efficacy in preventing wood colonization remains uncertain [49, 97, 99]. Reprogramming of flavonoid and phenylpropanoid pathways was also observed, involving the up-regulation of genes encoding for chalcone synthases (*CHS*) and phenylalanine ammonia-lyases (*PAL*). A comparison of the two symptomatic cultivars revealed that genes involved in the flavonoid and stilbenoid pathways were overexpressed in ‘Trincadeira’, confirming previous studies [87, 98, 99].

Hormonal metabolism was also reprogrammed in response to GTDs infection [16, 33, 87, 100]. The senescence-associated carboxylesterase 101 (*SAG101*) and enhanced disease susceptibility 1 (*EDS1*) genes, key regulators of innate immune responses against pathogen infection and the salicylic acid (SA) signalling pathway in *Arabidopsis* [101], were found to be significantly up-regulated in symptomatic ‘Trincadeira’ plants, confirming previous results during powdery mildew infection [35]. The SA signalling pathway plays a central role in local and systemic acquired resistance, leading to the activation of multiple defence mechanisms, including the production of PR proteins. During ETI, it has been proposed that SA-responsive genes are regulated by SA-independent mechanisms as well, thereby increasing the robustness of the innate immunity [102]. Nevertheless, it remains uncertain whether the tolerance of grapevine cultivars towards GTDs is contingent upon SA metabolism [64]. The jasmonic acid (JA) signalling pathway, typically linked to responses against necrotrophic pathogens, was demonstrated to be activated in cells surrounding the central SA-active cells around the infection sites [103]. While JA is often associated with localized defence responses, it can also contribute to systemic acquired resistance in conjunction with other signalling molecules, creating a state of heightened defence readiness throughout the plant [104]. It is proposed that this pathway may be activated in symptomatic ‘Trincadeira’ plants through the overexpression of genes associated with the oxylipin pathway (which encode lipoxygenases - *LOX* genes) in these plants, when compared with asymptomatic plants and symptomatic plants of ‘Alicante Bouschet’. Up-regulation of lipoxygenase genes was also observed in more tolerant cultivars in response to *Eutypa lata* [16], and JA-mediated signalling was activated upon trunk pathogen infection [87, 100], suggesting that these genes act in the regulation of phytohormones and defence responses.

AP2/ethylene-responsive transcription factors, known for their role in plant development and stress responses [105], were up-regulated in ‘Trincadeira’, aligning with their involvement in plant stress adaptation [106]. Ethylene-mediated responses are associated with xylem occlusion, as observed in response to *Xylella fastidiosa* infection [107], supporting its proposed role in grapevine responses to trunk pathogens [87, 88]. In recent years, some important transcription factor families have been associated with grapevine response to biotic stress [108–110], including trunk diseases [33, 88]. In the current research, we found that WRKY and heat shock transcription factors were enriched in symptomatic ‘Trincadeira’ plants, comparing with ‘Alicante Bouschet’. These results indicate that transcription factors may interact with SA, JA and ethylene to regulate the process of ‘Trincadeira’ tolerance to trunk pathogens.

Alongside these so-called classical defence responses, the host’s carbon metabolism undergoes substantial alterations in response to trunk pathogens, which is accompanied by a reduction in photosynthesis and the accumulation of hexoses in the apoplast, mainly in symptomatic plants [111]. Furthermore, recent studies have indicated that the regulation of carbon partitioning and competition for apoplastic sugars between the plants and the pathogens play a critical role in determining the outcome of the interaction [112], with both partners having their own sugar transport machinery [113]. It was already reported that invertase genes were strongly induced in grapevine tolerant cultivar ‘Merlot’ during *E. lata* infection [16]. However, our RNA-seq study did not reveal a coordinated up-regulation of the invertase genes *HT5* and *cwINV* in the tolerant ‘Trincadeira’ cultivar, although both genes were up-regulated by qPCR assays. *HT5* and *GIN2* were markedly overexpressed in ‘Trincadeira’, particularly in symptomatic plants, suggesting that they act during infection by facilitating the synthesis of compounds and energy necessary for pathogen response, and regulating sugar levels in vacuoles, which supports the production of defence-related metabolites and overall stress adaptation in grapevines. Conversely, *cwINV* was overexpressed in asymptomatic ‘Trincadeira’ plants, which may confer an advantage to the host by promoting the availability of sugar resources and/or precursors for defensive mechanisms. These results are in line with previously published information evidencing a role for these genes in molecular signalling cascades enhancing grapevine defence to different pathogens [114].

According to our results, the relative tolerance of ‘Trincadeira’ to this disease complex could be supported by the differential expression of a set of defence-related genes, including those related to sugar transport, and the activation of the stilbenoid, flavonoid, and phenylpropanoid pathways in symptomatic plants, even if it is not possible to counteract disease development. Moreover, synergistic interactions between the SA-, JA- and ethylene-mediated pathways allowed the grapevine to fine-tune its defence responses. These results suggest that the cv. ‘Trincadeira’ can perceive certain signals and initiate defence pathways in response to GTDs infection, which may contribute to its lower susceptibility to this disease complex. As previously demonstrated, ‘Trincadeira’ plants express R genes that are capable of recognising pathogen effectors and subsequently activating ETI. In this context, five disease resistance protein-encoding genes were up-regulated, with one in symptomatic and four in asymptomatic plants (such as *RPM1* and *RPS2*) when compared to ‘Alicante Bouschet’. The activated R genes regulate and activate proteases associated with programmed cell death, triggering the hypersensitive response and providing an efficient strategy to block pathogens [29, 86]. Therefore, our hypothesis is that R genes may act as upstream factors in the signalling pathway, activating innate immunity to restrict GTDs infection in this cultivar. In contrast, the susceptible cv. ‘Alicante Bouschet’ may have failed to trigger such defence responses, despite its capacity to detect the presence of GTDs-associated fungi.

### *PER42* is potentially involved in the inhibition of GTDs symptoms

Our results indicated that only two GO terms were significantly enriched in the overall comparison between GTDs symptomatic and asymptomatic plants, both related to the balance of the redox homeostasis: peroxidase activity and response to oxidative stress. In both cases, the sole gene that exhibited differential expression was *PER42*, which was overexpressed in asymptomatic plants. *PER42* encodes for an oxidoreductase enzyme involved in cell wall polysaccharide processes and plays an important role in the plant immune response to biotic stress [115]. In addition to their role in post-infection cell signalling, peroxidases can polymerise macromolecules, facilitating lignification and cell wall reinforcement, which increases resistance to mechanical damage, and, consequently, hinders pathogen invasion [116]. Peroxidases can also trigger the oxidative breakdown of phenolic compounds at the site of cell rupture caused by pathogens in the early stages of infection [117]. Additionally, plant peroxidases are expressed to restrict the spread of pathogen infection at the cellular level. This is achieved through the formation of structural barriers or the generation of a highly toxic environment through the production of ROS, which results in a programmed cell death at the site of infection [116, 118]. While ROS are important signalling molecules in plants, triggering the activation of defence-related genes and the production of antimicrobial compounds [118], their accumulation can lead to oxidative stress and cellular damage. Therefore, peroxidases and other enzymes contribute to maintaining a balanced ROS environment, enabling plants to swiftly respond to any stress without succumbing to oxidative damage [119].

Cell wall peroxidases and NADPH oxidase are known to mediate the oxidative burst, and evidence suggests that peroxidase enzymes might be an important component of plant disease resistance [120, 121]. In grapevine, peroxidases are involved in the response to *Diaporthe ampelina* [94] and contribute to resistance to *X. fastidiosa* [122], responsible for Pierse’s disease. Interestingly, peroxidase-expressing grapevines infected with *X. fastidiosa* can maintain high pathogen titres without displaying symptoms, suggesting a peroxidase-mediated dissociation between infection and symptom development [122]. This finding is significant for our study, as GTDs also affect the xylem vessels, and the absence of symptoms does not necessarily indicate the absence of trunk pathogens. Indeed, a previous study conducted by our research group revealed that asymptomatic plants hold some GTDs-associated fungi, albeit with a lower diversity than in symptomatic plants [37].

Lack of trunk diseases symptoms has been linked to the up-regulation of genes involved in photosynthetic electron transport and in the redox balance maintenance [123]. Given that peroxidases contribute to maintaining a balanced ROS environment, these data are consistent with our differential gene expression analysis, so we can propose that *PER42* is crucially involved in the grapevine tolerance mechanisms to GTDs by helping to maintain structural integrity, manage ROS levels and prepare the plant for defence against potential pathogens. These functions are critical for keeping plant health and resilience under various environmental conditions, and may explain the inhibition of GTDs symptoms, and therefore merit further research.

## Conclusions

The increasing public concern over the widespread use of chemical fungicides, together with the decreasing number of commercially available effective fungicides and the emergence of trunk diseases raise serious doubts about the future of worldwide viticulture. Furthermore, the complex signalling network and defence pathways involved in this pathosystem reveal the intricacies of the grapevine response to GTDs, which in turn elucidates the reasons behind the lack of effective protection practices to control these diseases. To the best of our knowledge, most of the transcriptomic studies on grapevine response to GTDs were performed using plants inoculated with specific GTDs-associated fungi, under controlled conditions. However, field-based studies (such as this work) introduce additional complexity due to the interaction of multiple fungi and abiotic stresses, which may influence gene expression patterns. This inherent variability represents a challenge when isolating the specific molecular responses triggered by pathogen infection. Additionally, a constraint of transcriptome analysis is that some DEGs may be activated as secondary responses to stress factors not directly related to infection, potentially complicating the identification of key players in grapevine defence. Despite these challenges, our study provides a more complete understanding of the molecular response mechanisms, which could ultimately lead to more effective breeding strategies and more sustainable disease management practices. Further studies on multi-omics profiling to study the metabolic responses that occur during infection, will allow the discovery of key metabolites, biochemical pathways and molecular interactions involved in plant defence and fungal virulence.

The outcomes of this study contribute to a better understanding of the regulatory networks of the grapevine response to GTDs and indicates useful candidate genes for functional validation, which have broad potential for their use in engineering pathogen resistance as promising candidates for sustainable grapevine plant breeding. These candidates might be exploited through marker-assisted selection or integrated into genome editing approaches, such as CRISPR/Cas9, to develop cultivars with enhanced resistance to trunk diseases. By leveraging these genomic tools, breeders can accelerate the development of grapevine cultivars that are both resilient and sustainable, ultimately improving vineyard productivity and reducing reliance on chemical control methods.

Undoubtedly, the use of tolerant cultivars represents an intriguing and sustainable approach to the management of GTDs infections. Nevertheless, it is becoming increasingly evident that no single efficacious control measure should be employed to control GTDs. It is therefore necessary to adopt an integrated approach to disease management, combining different control methods from the nursery stage, through the planting of new vineyards and into the production phase. Further research is still required to explore the microbiome associated with symptomatic and asymptomatic GTDs plants. These approaches represent potential avenues for future exploitation and have the potential to contribute significantly to the development of more sustainable and specific alternative control methods for trunk diseases.

## Electronic supplementary material

Below is the link to the electronic supplementary material.

Supplementary Material 1

Supplementary Material 2

Supplementary Material 3

Supplementary Material 4

Supplementary Material 5

Supplementary Material 6

Supplementary Material 7

Supplementary Material 8

Supplementary Material 9

Supplementary Material 10

## Data Availability

The datasets generated and analysed in this study have been deposited in NCBI’s Gene Expression Omnibus and are available through the GEO Series accession number GSE283169 (https://www.ncbi.nlm.nih.gov/geo/query/acc.cgi?acc=GSE283169), or from the corresponding author on reasonable request.
