# Supplementary material for: Transcriptome profiling of symptomatic vs. asymptomatic grapevine plants reveals candidate genes for plant improvement against trunk diseases
Source: BMC Plant Biol. 2025 Jul 2;25:811. doi: 10.1186/s12870-025-06763-9 (PMC12220349; doi:10.1186/s12870-025-06763-9)
Supplement: Supplementary file 1 — Supplementary Material 1 [file 12870_2025_6763_MOESM1_ESM.docx]

**Supplementary Table S1.** Primer design for qPCR.

| **Genes** | **ID** | **Sequence (5'-3')** | **Product Size (bp)** | **NCBI Reference Sequence** | **Efficiency (%)** | **R^2^** | **Reference** |
| --- | --- | --- | --- | --- | --- | --- | --- |
| ***GAPDH*** | VIT_00007521001 | FW: CCACAGACTTCATCGGTGACA  RV: TTCTCGTTGAGGGCTATTCCA | 70 | XM_002263109.3 | 100.53 | 0.9996 | [16] |
| ***PEP*** | VIT_00020705001 | FW: CCTCCTCCTCCAGATTGC  RV: GGCTTGCTTGATTCCATTATC | 198 | AF236126.1 | 100.91 | 0.9997 | [53] |
| ***UBC*** | VIT_00027045001 | FW: CATAAGGGCTATCAGGAGGAC  RV: TGGCGGTCGGAGTTAGG | 161 | EE253706 | 100.89 | 0.9993 | [53] |
| ***PR2*** | VIT_00035013001 | FW: GCAGTCGGGAACGAAGTGAG  RV: ATGGAGGGTAGGAGTTGCCC | 172 | NM_001280967.2 | 105.12 | 0.9973 | [16] |
| ***LOX*** | VIT_00000083001 | FW: TGCTCTACCCCACAAGCGAA  RV: AGCAGTGTGCTCATGATTTTCCAG | 95 | NM_001281249.1 | 107.96 | 0.9984 | [16] |
| ***STS1*** | VIT_00010561001 | FW: AGGGAAGCAGCATTGAAGGC  RV: CGGGCATTTCTACACCGGAG | 97 | XM_002263845.4 | 104.03 | 0.9996 | [16] |
| ***HT5*** | VIT_00017937001 | FW: TAGTGATGCGTCCCTCTACTC  RV: CTTCCAGCAAGAGCAATCGAC | 113 | NM_001281278.1 | 99.13 | 0.9982 | [16] |
| ***cwINV*** | VIT_00016869001 | FW: ACGAATCATCTAGTGTGGAGCAC  RV: CTTAAACGATATCTCCACATCTGC | 236 | NM_001281279.1 | 93.42 | 0.9538 | [16] |
| ***GIN2*** | VIT_00001272001 | FW: GGGGTTCTTCATGCTGTCCC  RV: TGCTTGACTCCGGGACCATT | 116 | XM_019217574.1 | 113.66 | 0.9950 | [16] |
| ***PER42*** | VIT_00012727001 | FW: CTTGTGAGAGGTATGAAGATG  RV: ACCATAACGCCATTGTAAC | 193 | XM_002274733.3 | 97.27 | 0.9997 | [49] |
| ***MAPKKK17*** | VIT_00030452001 | FW: ACCTTAGGCTCTGGCTCCTC  RV: CACACCCCTTGTAGCCAACT | 169 | XM_002269624.3 | 99.22 | 0.9994 | [52] |
| ***PR1*** | VIT_00037005001 | FW: GCAACTATATCGGACAACGTCCTT  RV: TCACCATGCTCTAACAGTACCCA | 80 | XM_002273752 | 94.82 | 0.9991 | [50] |
| ***PR4*** | VIT_00036279001 | FW: GCCCAGAGCGCCAGCAATGT  RV: CGCCATGCCAAGGGCTTGCT | 125 | XM_002264684 | 92.96 | 0.9994 | [50] |
| ***TLP8*** | VIT_00019840001 | FW: TCCTGGAGCCTGACTGTGAAC  RV: GGGCAGAAGATAACATCATAGTTG | 515 | XM_002282994 | 85.17 | 0.9997 | [51] |
| ***TLP3*** | VIT_00019835001 | FW: CCCTGGCACAACTGGAG  RV: GGCAGAAGATAACTTCATAGTTGG | 494 | XM_010662912.2 | 106.12 | 0.9917 | [51] |
| ***HSP101*** | VIT_00007880001 | FW: AATGAGACTCTTGCTGGGGC  RV: CAGCACCGATTATGGCTTGC | 130 | NM_001280893.1 | 103.26 | 0.9988 | [52] |
| ***bHLH94*** | VIT_00000012001 | FW: CATCATCTTCCTCTCGCCCC  RV: CTCATTCATCTGCTTGCGGC | 122 | XM_002281047.4 | 102.22 | 1.0000 | This study |
| ***SAUR71*** | VIT_00036807001 | FW: AGCGCCGAGTTTCTTAACCA  RV: AGTGCTTCAAGGACTCGCTC | 134 | XM_002266212.4 | 108.76 | 0.9973 | This study |
| ***MYB61*** | VIT_00019410001 | FW: GAGCCAACTGGACATGGTGA  RV: GATGCTGGAGGAGGTAGTGC | 222 | XM_002272968.3 | 95.07 | 0.9979 | This study |

*bHLH94*, *SAUR71* and *MYB61* primers were designed using Primer3 software version 0.4.0 from the specific sequence of *Vitis vinifera* deposited in the NCBI GenBank.
