# Supplementary material for: Transcriptome profiling of symptomatic vs. asymptomatic grapevine plants reveals candidate genes for plant improvement against trunk diseases
Source: BMC Plant Biol. 2025 Jul 2;25:811. doi: 10.1186/s12870-025-06763-9 (PMC12220349; doi:10.1186/s12870-025-06763-9)

**Supplementary Figure S1.** Principal Component Analysis (PCA) according to the gene expression of the sample groups. The first principal component is plotted on the x-axis and the second principal component is plotted on the y-axis. The value after the principal component label indicates the amount of variance explained by each principal component.


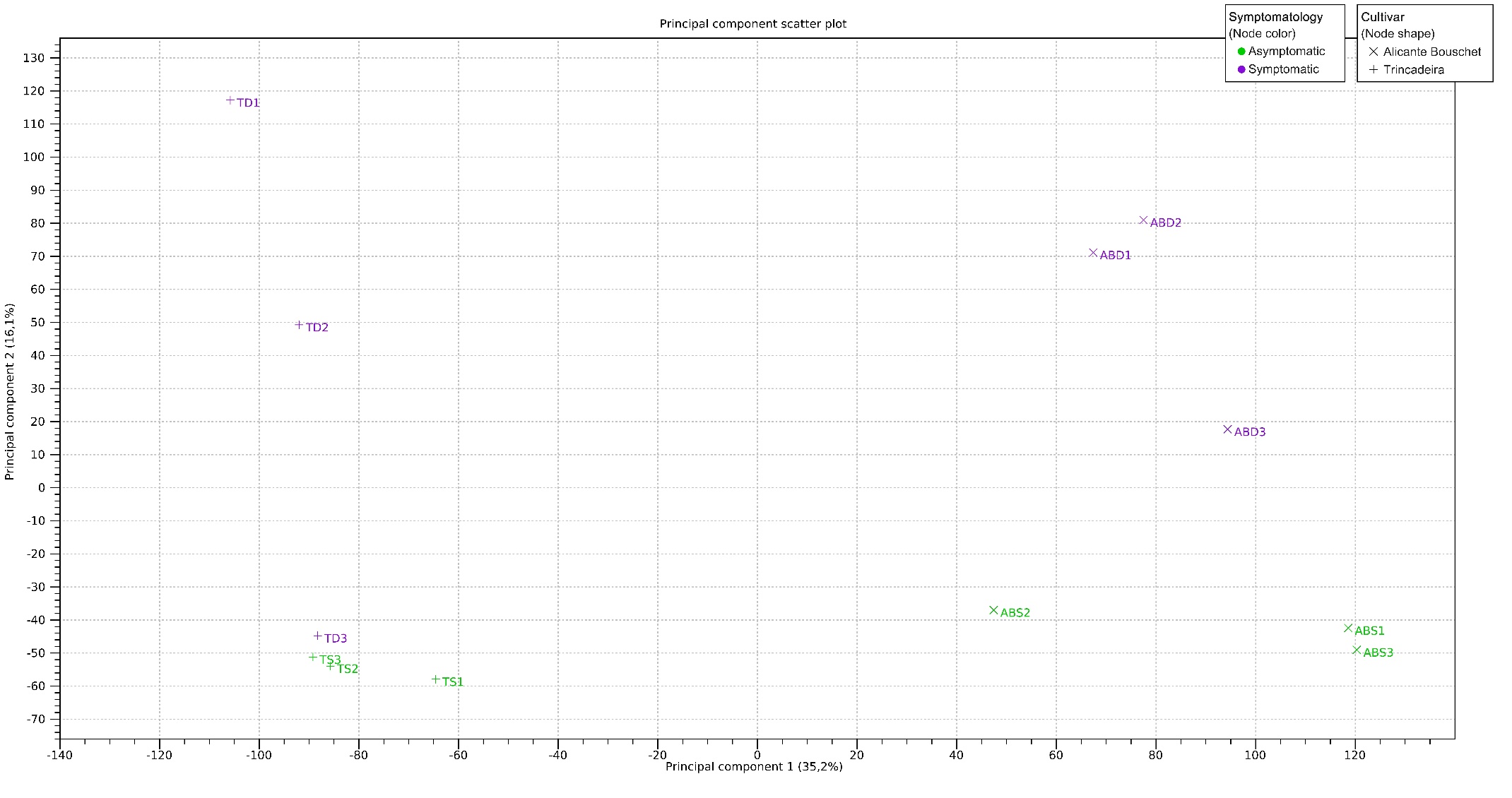

Supplement: Supplementary file 4 — Supplementary Material 4 [file 12870_2025_6763_MOESM4_ESM.docx]
