# Supplementary material for: Transcriptome profiling of symptomatic vs. asymptomatic grapevine plants reveals candidate genes for plant improvement against trunk diseases
Source: BMC Plant Biol. 2025 Jul 2;25:811. doi: 10.1186/s12870-025-06763-9 (PMC12220349; doi:10.1186/s12870-025-06763-9)

**Supplementary Figure S2.** Volcano plots of the six comparison groups of GTDs symptomatic (symp.) and asymptomatic (asymp.) plants from cv. ‘Alicante Bouschet’ (AB) and ‘Trincadeira’ (T) containing the differentially expressed genes (FDR of ≤ 0.05 and fold change of ≥ 2.0 or ≤ -2.0), indicated in red colour. Blue dots correspond to genes that are not differentially expressed.

T *vs* AB:


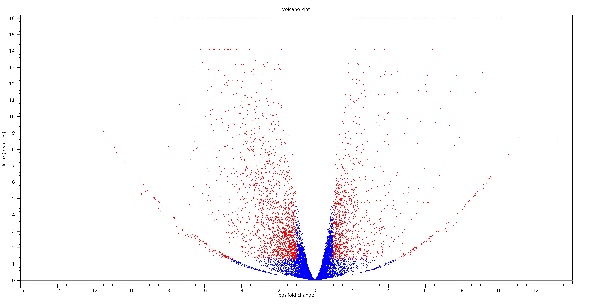


symp. *vs* asymp.:


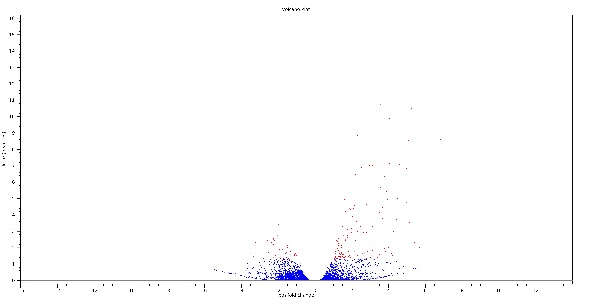


AB asymp. *vs* T asymp.:


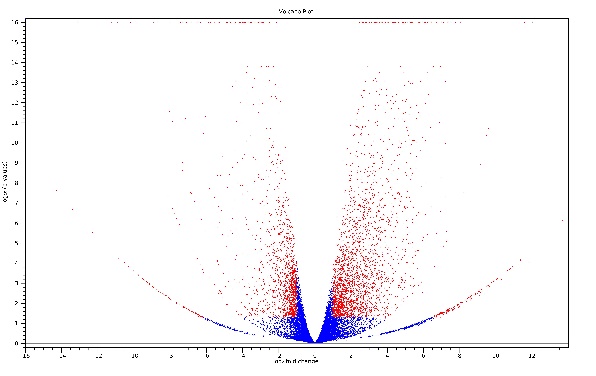


AB symp. *vs* AB asymp.:


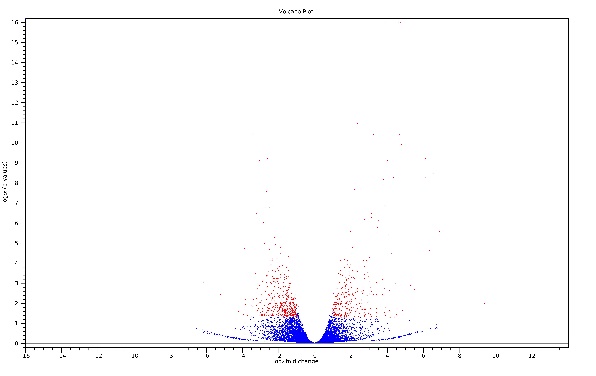


AB symp. *vs* T symp.:


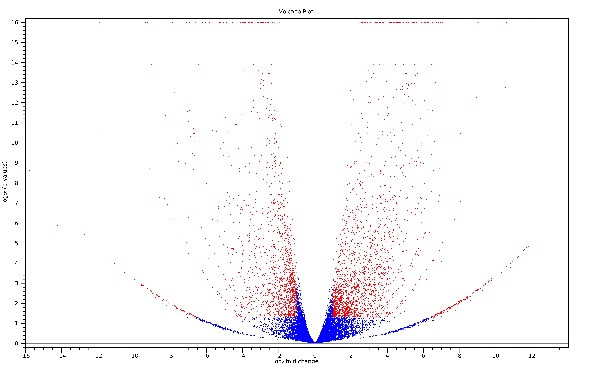


T symp. *vs* T asymp.:


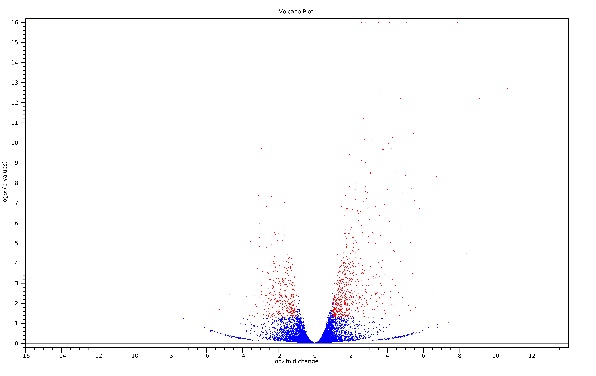

Supplement: Supplementary file 6 — Supplementary Material 6 [file 12870_2025_6763_MOESM6_ESM.docx]
