# Supplementary material for: Transcriptome profiling of symptomatic vs. asymptomatic grapevine plants reveals candidate genes for plant improvement against trunk diseases
Source: BMC Plant Biol. 2025 Jul 2;25:811. doi: 10.1186/s12870-025-06763-9 (PMC12220349; doi:10.1186/s12870-025-06763-9)

**Supplementary Figure S3.** Hierarchical clustering and heat map of differential gene expression analysis across all samples. The red colour represents overexpression, while the blue colour represents underexpression.


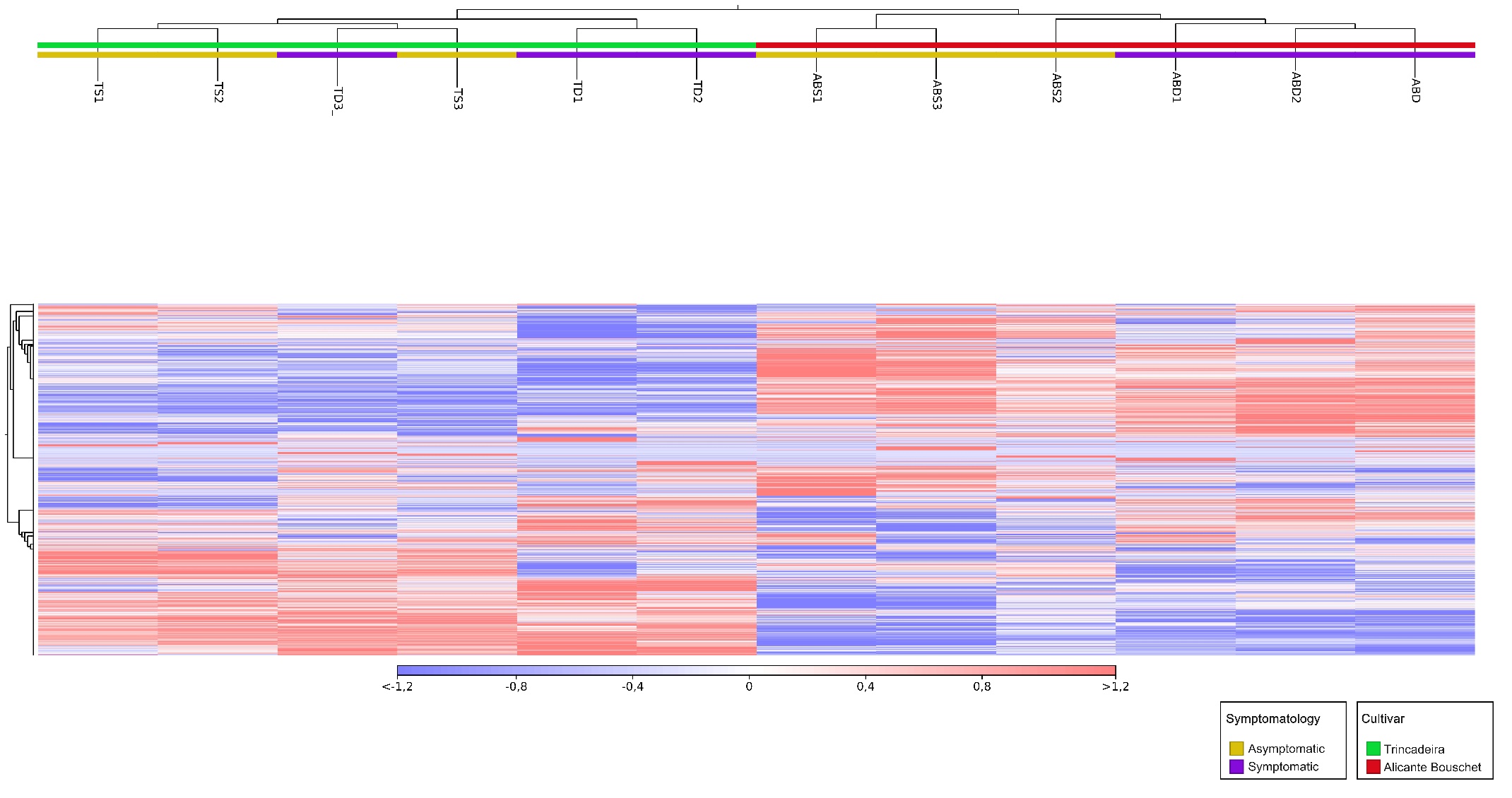


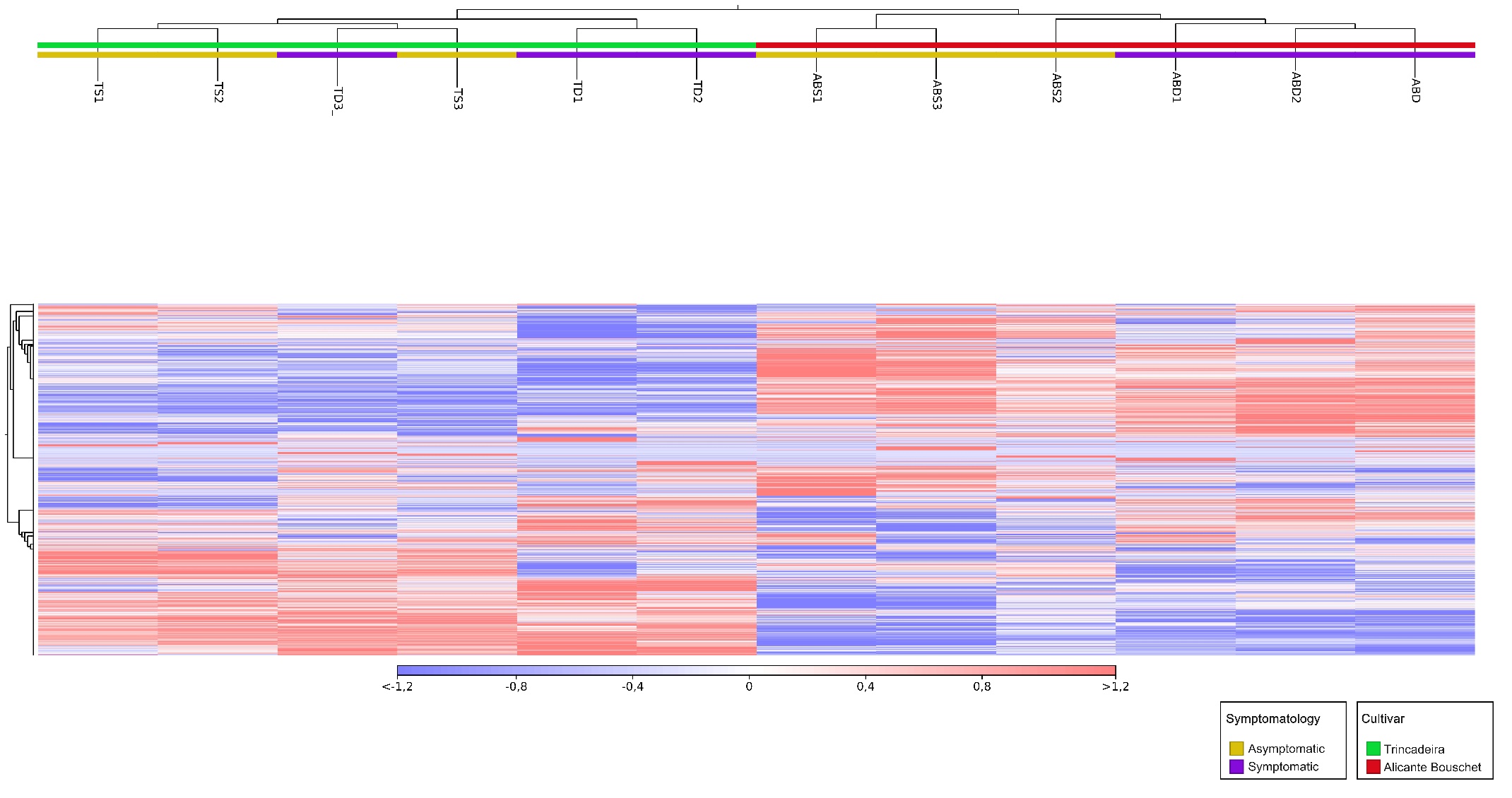

Supplement: Supplementary file 7 — Supplementary Material 7 [file 12870_2025_6763_MOESM7_ESM.docx]
