# Supplementary material for: Transcriptome profiling of symptomatic vs. asymptomatic grapevine plants reveals candidate genes for plant improvement against trunk diseases
Source: BMC Plant Biol. 2025 Jul 2;25:811. doi: 10.1186/s12870-025-06763-9 (PMC12220349; doi:10.1186/s12870-025-06763-9)
Supplement: Supplementary file 8 — Supplementary Material 8 [file 12870_2025_6763_MOESM8_ESM.docx]

**Supplementary Figure S4.** Relative gene expression of 16 genes through qPCR. Represented values are means of relative gene expression with their respective standard errors represented by vertical bars. Significance: *** *p* < 0.001, ** *p* < 0.01, * *p* < 0.05 and 0.05 < *p* < 0.10 considered as trends.

**Cultivar (T *vs* AB):**


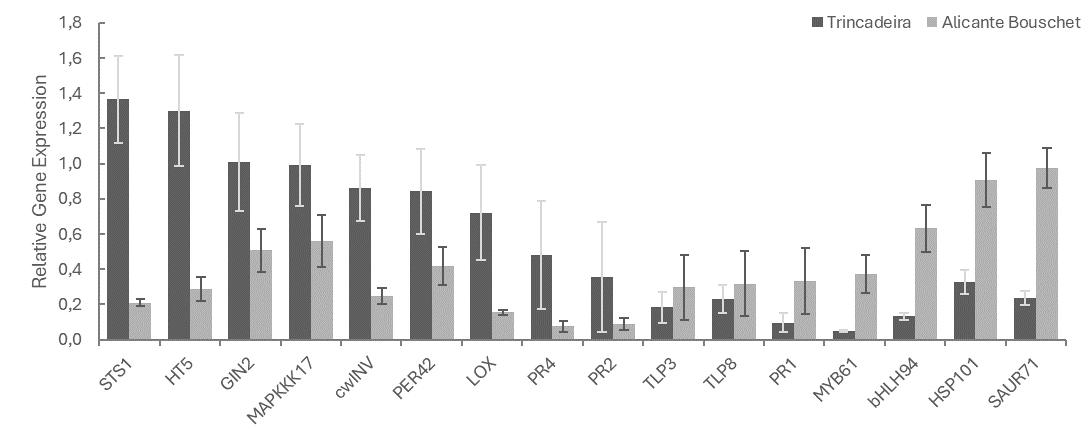


*

*

*

**

**

**

***

*p* = 0.075

**Symptomatology (symp. *vs* asymp.):**


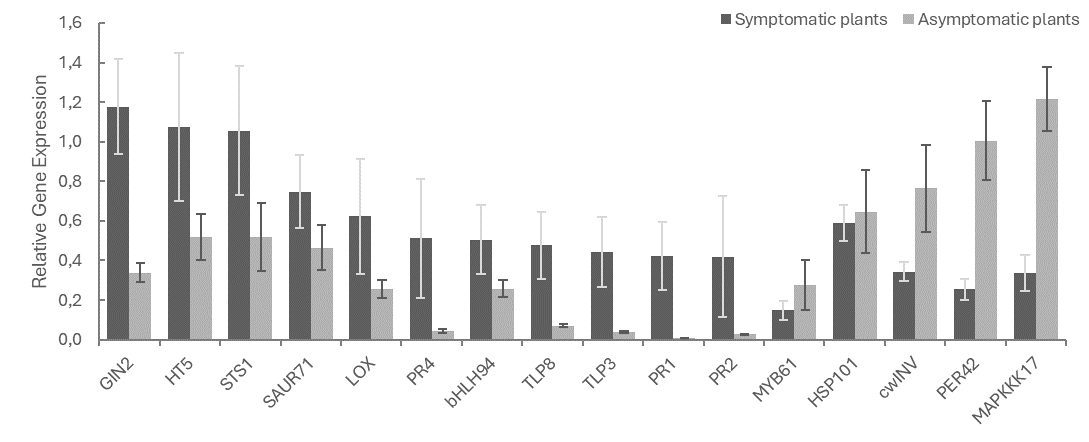


*

*

**

**

***

*p* = 0.103

*p* = 0.054
