# Supplementary material for: Transcriptome profiling of symptomatic vs. asymptomatic grapevine plants reveals candidate genes for plant improvement against trunk diseases
Source: BMC Plant Biol. 2025 Jul 2;25:811. doi: 10.1186/s12870-025-06763-9 (PMC12220349; doi:10.1186/s12870-025-06763-9)

**Supplementary Figure S5.** Pearson correlation between gene expression data obtained by qPCR and RNA-seq data for two comparison groups.

**Cultivar (T *vs* AB):**


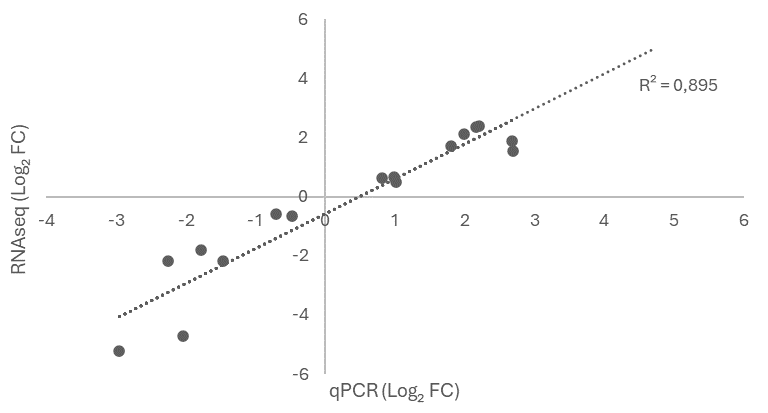


**Symptomatology (symp. *vs* asymp.):**


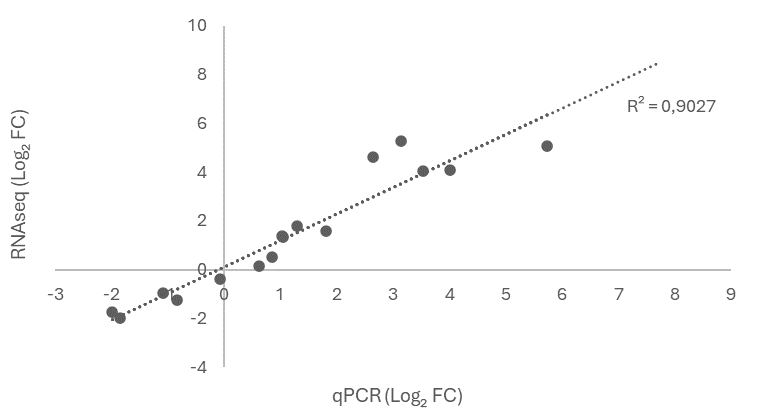

Supplement: Supplementary file 9 — Supplementary Material 9 [file 12870_2025_6763_MOESM9_ESM.docx]
